# Challenges in Patient Blood Management for Cardiac Surgery: A Narrative Review

**DOI:** 10.3390/jcm10112454

**Published:** 2021-06-01

**Authors:** Valentina Rancati, Emmanuelle Scala, Zied Ltaief, Mohamed Ziyad Gunga, Matthias Kirsch, Lorenzo Rosner, Carlo Marcucci

**Affiliations:** 1 Department of Anesthesiology, University Hospital of Lausanne, Rue du Bugnon 46, 1011 Lausanne, Switzerland; Emmanuelle.scala@chuv.ch (E.S.); Lorenzo.rosner@chuv.ch (L.R.); carlo.marcucci@chuv.ch (C.M.); 2 Department of Intensive Care Medicine, University Hospital of Lausanne, 1011 Lausanne, Switzerland; zied.ltaief@chuv.ch; 3 Department of Cardiac Surgery, University Hospital of Lausanne, 1011 Lausanne, Switzerland; Ziyad.gunga@chuv.ch (M.Z.G.); Matthias.kirsch@chuv.ch (M.K.); 4 Faculty of Biology and Medicine, University of Lausanne, 1011 Lausanne, Switzerland

**Keywords:** cardiac surgery, anemia, transfusion, patient blood management

## Abstract

About 15 years ago, Patient Blood Management (PBM) emerged as a new paradigm in perioperative medicine and rapidly found support of all major medical societies and government bodies. Blood products are precious, scarce and expensive and their use is frequently associated with adverse short- and long-term outcomes. Recommendations and guidelines on the topic are published in an increasing rate. The concept aims at using an evidence-based approach to rationalize transfusion practices by optimizing the patient’s red blood cell mass in the pre-, intra- and postoperative periods. However, elegant as a concept, the implementation of a PBM program on an institutional level or even in a single surgical discipline like cardiac surgery, can be easier said than done. Many barriers, such as dogmatic ideas, logistics and lack of support from the medical and administrative departments need to be overcome and each center must find solutions to their specific problems. In this paper we present a narrative overview of the challenges and updated recommendations for the implementation of a PBM program in cardiac surgery.

## 1. Introduction: The Scope of the Problem

Cardiac surgery is a generic term for various types of major surgery performed on the heart and great vessels, usually performed on cardiopulmonary bypass (CPB) with full systemic anticoagulation. CPB has detrimental effects on hemostasis, resulting in dilution and consumption of clotting factors, platelets activation and hyperfibrinolysis that persist after reversal of the heparin effect by protamine [1].

The risk of significant blood loss and subsequent allogenic transfusion is therefore higher than for other types of surgery. Between the years 1999–2010 blood product utilization in the US for cardiac surgery increased, despite the decline in number of cardiac operations. Indeed, the percentage of patients transfused increased from 12.3% in 1999 to 34% in 2010 [2].

In the same period, the Multicentric Study of Perioperative Ischemia Epidemiology II investigated transfusion practice worldwide, involving centers in the USA, Canada, Europe and Asia. A large variability between countries, a potential indicator for sub-optimal quality, emerged from the study, suggesting the need for an international standardization of perioperative transfusion practice [3].

More recently, a report from the Society of Thoracic Surgery, covering the period 2009–2017, reported a negative trend in the use of blood products for cardiac surgery; nonetheless, the rate of patients transfused remains high, ranging from about 30% for isolated mitral valve repair to 80% for combined mitral valve and CABG surgery. [4]

Although definitive evidence from large prospective trials is lacking, transfusion of blood products is strongly associated with adverse outcomes and efforts to reduce transfusion requirements are strongly recommended [5,6,7]. These include identifying and correcting preoperative risk factors for transfusion such as anemia and antithrombotic therapy, optimizing perioperative hemostasis and using blood products on an evidence based and patient specific rationale.

In 2005 the hematologist J. Isbister used the term “Patient Blood Management” (PBM) to propose a paradigm shift that placed the patient and his needs at the center of a multi-disciplinary approach aiming to ensure the best outcome in opposition to blood product-related transfusion medicine, more focused on safety and quality of blood products [8].

The World Health Organization (WHO) endorses PBM since 2010, with reference to the following three-pillars concept: “before surgery every reasonable measure should be taken to optimise the patient’s own blood volume, to minimise the patient’s blood loss and to harness and optimize the patient-specific physiological tolerance of anaemia”. (WHA 63.12) [9].

In the ensuing decade, PBM programs have been implemented in Europe, the United States [10,11,12,13] and, most successfully, in Western Australia where the National Blood Authority published extensive guidelines on the implementation of a nationwide PBM program [14]. 

In 2017, the European Commission wrote a guide to assist Health Authorities in building national programs of PBM, encouraging the development of large government driven PBM initiatives and giving a practical framework for the implementation of high quality PBM programs [15]. Extensive PBM bundles have been published to implement or improve PBM measures in a single institution, covering all the aspects of the strategy [16].

Finally, during the ongoing COVID-19 pandemic, the International Foundation of Patient Blood Management (IFPBM) and the Society for the Advancement of Blood Management (SABM) group called for action for implementing PBM programs in a context when the access to blood products is even more limited than usual [17].

Despite the general agreement on the benefits to be expected, starting a PBM program in the context of cardiac surgery presents specific challenges. In this perspective statement we aim to describe the specific organizational, logistical and medical requirements and to identify and prevent some of the most common pitfalls when establishing an institutional PBM program. 

Table 1 resumes the elements and recommendations that constitute the basis of the three pillars on patient blood management in cardiac surgery.

## 2. Challenges in Building an Adequate PBM Structure

The PBM structure should comprise staff from all disciplines involved in transfusion on the medical, nursing and administrative levels. 

### 2.1. PBM Coordinator

The key position is held by the PBM coordinator who has the ardent task of organizing the training of staff, defining the logistical requirements, establishing clinical pathways and reporting to the hospital administration.

Generally, the PBM coordinator has professional qualification in a specific area of expertise (anesthesia, transfusion medicine, hematology, etc.). For a successful program, the coordinator must have strong leadership skills, “see the big picture” and connect the dots of different stakeholders to create new synergies. 

The implementation of a PBM program requires major changes to institutional practices and organization. In this process, the response of human resources plays a paramount role; people cannot simply be asked to change. Indeed, PBM implementation may overturn well-rooted habits and practices. In such a learning process, the coordinator must become a trusted guide, capable of overcoming personal barriers with the aim of building a strong “teamwork” attitude in a group of people sharing the same culture and objectives. 

Beside the human factor, a PBM coordinator must deal with procedure planning and their financial coverage. This requires a blend of expertise in the medical aspects, familiarity with the workflow and its logistic requirements, skills in cost analysis and budget planning [16].

### 2.2. Stakeholders in PBM Applied to Cardiac Surgery 

Due to the complexity, the invasive character and the technical aspects of cardiac surgery, PBM involves stakeholders at multiple levels. 

In the prehospital phase, general practitioners and cardiologists are responsible for the detection and correction of anemia, the management of antithrombotic drugs and the optimization in the treatment of comorbidities.

In the operation theatre, cardiac anesthesiologists, cardiothoracic surgeons and perfusionists are major players in minimizing blood loss. Intensive care specialists take over in the immediate postoperative period, optimizing hemostasis and the oxygen delivery/consumption balance, according to the patient’s tolerance to anemia and through the application of appropriate transfusion triggers. Clinical hematologists and transfusion medicine specialists may be involved to manage complex hemostatic disorders. Nurses are pivotal in timely bedside problem detection. Fast and reliable responses to clinical findings and pathologic hemostasis and chemistry laboratory results is a prerequisite. 

Lack of knowledge or motivation in one of these groups, or lack of coordination between groups may jeopardize the whole PBM project. To manage such a multilevel process, the PBM coordinator should be assisted by a dedicated committee, including one leader from each stakeholder group. PBM also has external stakeholders: patient’s advocacy groups and opinion leaders may be involved in the decisional processes and strategy development; epidemiologists have a central role in the outcome evaluation necessary to feedback-guided management. 

### 2.3. Funding

Unless PBM implementation is prioritized by institutional policies, the PBM coordinator needs to deal with hospital administrators to obtain the necessary resources. In the long run, PBM is cost effective [18,19,20,21].

Nonetheless, economic resources need to be allocated and maintained until the breakeven point is achieved. 

As mentioned above, the human factor is central to PBM. PBM, in turn, is very demanding for its stakeholders and loss of team members “en route” may be a major problem, with both health and economic impact. Accordingly, protected working time, incentives and career perspectives for nurses and physicians involved in PBM development and implementation are also important keys to success.

### 2.4. Education

PBM is rarely a part of current teaching programs in medical school and at the postgraduate level. The lack education on the subject in the public at large, the individual patients and the health professionals is identified as one of the main obstacles to establishing PBM in daily clinical practice. Government bodies and scientific societies strongly encourage education on pre- and postgraduate levels. 

Carefully designed instruments should also be made available to inform patients about the risk/benefit ratio of transfusion therapy and on alternatives potentially available. Education is fundamental to achieve the necessary change in culture and to correct erroneous a priori concepts and dogmas on transfusion practices.

### 2.5. Quality Monitoring

Quality monitoring should be a concern form the onset of a PBM program in any given institution. 

Procedural efficiency, outcomes and quality indicators should be monitored to create a database for internal and external benchmarking. 

## 3. Overcoming Resistances to a Change

A PBM program should be considered a major change. Resistance to change is part of human behavior. In the Lewin’s cornerstone model for understanding and carrying out an organizational change three stages are identified: unfreezing, moving and refreezing. In the first phase, the team is prepared for the change, in the second the change is implemented, in the third the change is consolidated and becomes the standard for common practice. Ref. [22] Resistance to change is prone to occur especially in the first and second stage. 

Analysis of physicians’ compliance to guidelines has led to the identification of common reasons for deviation [23]:

- Lack of knowledge or incorrect outcome expectancy: the physician simply ignores the recommendation or does not believe that it leads to a better outcome.
- Inertia in abandoning well rooted practice: this may result from active choices, motivated by skepticism or fear, or be simply intellectual idleness.
- Protocols may compromise “autonomy” of physicians: adherence to protocols may be perceived as a severe limitation of professional freedom, affecting self-perception and status.
- External barriers: inability to reconcile patient preferences with recommendations, lack of time or resources, organizational constraints.

The strategy to fill knowledge gaps is education and communication; inertia can be overcome by facilitation and support (i.e., additional training, mentoring); and participation/involvement is often the best way to bring on-board who would otherwise resist even after adequate education has been provided. If involved in the decisional process, people do not feel overruled and are more likely to accept it; moreover, involvement implies to take responsibilities and merit in the action. 

## 4. Challenges in Preoperative Anemia Correction: Barriers and Misconceptions

In a recent review the prevalence of preoperative anemia in adult cardiac surgery is estimated between 20 and 30% [24,25].

The negative impact of preoperative anemia on outcome is well-known and documented by a vast body of literature [26,27,28,29].

Preoperative anemia predicts the need for red blood cells transfusion; it affects cardiac and renal morbidity and post-operative mortality. 

### 4.1. Anemia, Cardiac Surgery and Outcome in Women 

According to the WHO, mild anemia is defined as Hb < 120 g/L for women and <130 g/L for men, and severe anemia as Hb < 100 g/L for both genders. Notably, the suggested Hb cut-off value is lower in women; nonetheless, this may contribute to gender differences in cardiac surgery outcome. Ref. [30] Women are more likely to receive allogenic blood transfusion and larger quantities of blood than men. This is not surprising, since the circulating blood volume is lower in women. For the same procedure, perioperative bleeding (in absolute terms) is similar between genders; thus, the same blood loss represents a higher percentage of total circulating volume in females than in males. For the same reason, the hemodilution caused by the priming volume of the extracorporeal circulation circuit is more pronounced in women, thus aggravating intra- and postoperative coagulation derangements.

As expected, the higher rate of transfusion in women is associated with increased mortality when compared to male patients matched for other factors [31,32]. 

Gender-specific anatomical factors, such as smaller arteries and restricted access to total arterial revascularization (increasing the risk of residual ischemia after CABG), lower body surface area and smaller annular size (complicating valvular surgery), account for the poorer outcome of cardiac surgery in females. Nonetheless, the larger impact of preoperative anemia may independently contribute to it. Ref. [33] Therefore, in accord with other authors [24,30], we have adopted the higher cut-off value (Hb 130 g/L) for both genders.

### 4.2. When to Start Anemia Treatment?

The organization of, and the resources needed for, the screening and treatment of preoperative anemia are possibly more complex than the clinical management itself. First, blood samples have to be taken and analyzed, the results have to be checked, the patient has to be informed of the results and a treatment schedule needs to be proposed. For some types of anemia further specialist advice or investigations need to be organized. Treatment then has to be administered and treatment success needs to be confirmed before surgery. Meanwhile the index surgery needs to be planned, but the surgeons schedule should be flexible enough to permit further delays if supplemental treatment is warranted. 

The specific organization of this “ferrous pathway” depends on the local human and logistical resources available, on the existence and level of efficacy of a regional health network including family physicians, specialists, laboratories, ambulatory clinics and the surgical center, and last but not least, on the motivation of all parties and their conviction of the importance of such a program. 

The treatment of preoperative anemia and iron deficiency should commence as early as possible, ideally as soon as the decision to undertake surgery is made. Ref. [34] In our center we try to implement the referring physician to screen for anemia and have lab results available at the first surgical consultation where the indication to surgery is made. The intervention can then be scheduled according to the time needed to treat anemia if present. 

Several screening and treatment algorithms have been published and can be easily used as such or adapted to the specific need and limitations of any center. An in-depth discussion of their rationale and scientific value lies beyond the scope of this review.

**Figure 1:**
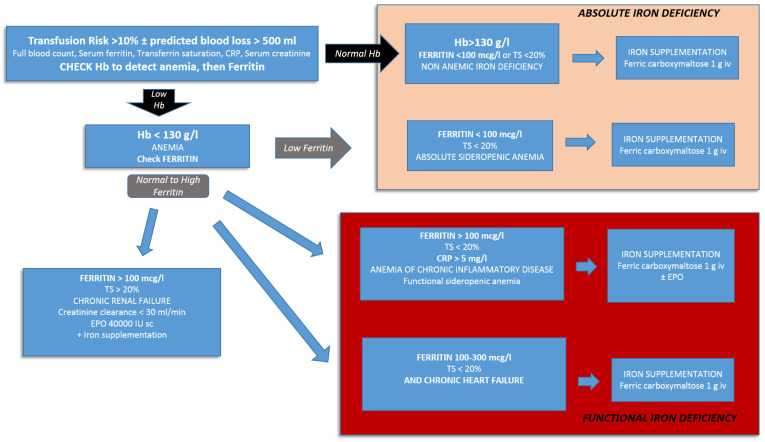
Algorithm for anemia management in the cardiac surgical population. CRP = serum C-reactive protein, Hb = hemoglobin, TS = transferrin saturation, EPO = Erythropoietin. Note: the higher ferritin cut-off of 100 mcg/L is used to identify absolute iron deficiency since the high prevalence of an inflammatory state in the cardiac surgical population.

In Figure 1 we present a simplified algorithm specifically adapted for the cardiac surgical population.

In cardiac surgery, the time between an acute cardiac event, the surgical indication and the actual intervention may be short. However, is it too short to start treating preoperative anemia? Actual evidence suggests that this may not be the case.

In 2011 a first small trial demonstrated that the need of perioperative RBC transfusion may be reduced in anemic patients by the administration of a single dose of EPO, associated to parenteral iron supplementation, on the day before valvular heart surgery. Ref. [35] In 2019 a larger single center, randomized, controlled trial demonstrates that a combined administration of intravenous iron, EPO, B12 vitamin and folic acid on the day before cardiac surgery, significantly reduced the risk of RBC transfusion in patients with overt preoperative anemia or isolated iron deficiency. Ref. [36] The authors did not find a reduction of exposure to blood products, 44% of patients were transfused in the treatment group compared 53% in the placebo group. Yet, the median number of RBC units transfused per patient was significantly lower in the treatment group while the Hb concentration, reticulocyte count and reticulocyte Hb content were all higher. Their treatment schedule was successful in reducing transfusion requirements but came with a more than twofold increase in treatment related costs from a mean of CHF 480 to 1052.

## 5. Iron Deficiency: An Emerging Marker for Worse Outcome and a Target for Treatment

Iron deficiency is the second leading cause of anemia (after iatrogenic anemia in hospitalized patients), accounting for about 30% of anemic patients undergoing cardiac surgery. Refs. [25,37] Iron deficiency can be described as “absolute”, in the presence of reduced iron stores in the liver and reticuloendothelial system, or “functional”, if iron stores are preserved but difficult to mobilize. The latest is typical of inflammatory chronic disease. 

Iron plays an important role in hematopoiesis, in muscle metabolism, immune system, and it is a cofactor for mitochondrial respiratory chain proteins. Ref. [38] There is evidence that iron deficiency is associated with a worse outcome in critically ill patients [39], chronic heart failure patients [40] and, recently, in cardiac surgical patients [41].

True (absolute) iron deficiency is strongly predicted by a serum ferritin level < 30 μg/L [42].

In the presence of inflammation (CRP > 5 mg/L), as often is the case in cardiac surgery population, a higher cut-off value for ferritin < 100 μg/L and/or TSAT < 20% strongly suggests absolute iron deficiency. In such patients, hematopoiesis could be insufficient in the postoperative period if surgical blood loss is moderate-to-high increasing the risk of developing severe postoperative anemia. 

Finally, ferritin > 100 μg/L along with TSAT < 20% indicates a functional iron deficiency, with iron sequestration in tissues. This may be seen in critically ill patients, inflammatory conditions and after treatment with erythropoiesis stimulating agents [34].

There is consensus between experts recommending intravenous iron over oral iron supplementation in anemic iron deficient patients undergoing major surgery when the delay is less than 6 weeks [34]. The dose of parenteral iron required to achieve the target Hb is usually between 1000 et 1500 mg and can be calculated from Ganzoni’s formula [43].

With suitable dosage of parenteral iron, an increase in Hb of 10–20 g/L after 2–4 weeks may be expected [24]. 

## 6. Are Erythropoiesis Stimulating Agents (ESA) Effective and Safe?

According to EACTA/EACTS 2017 Guidelines on PBM in cardiac surgery [7], erythropoietin with iron supplementation should be considered to reduce post-operative transfusion in patients with non-iron deficiency anemia (e.g., EPO, vitamin D or folate deficiency) undergoing elective cardiac surgery (Class of recommendation IIb, level of evidence C). The low level of evidence was derived from three RCTs [35,44,45].

After release of the European guidelines, erythropoietin (40,000 UI s.c.) was administered along with parenteral iron supplementation in patients undergoing cardiac surgery, in two studies: the prospective RCT by Spahn et al. [36] mentioned earlier and a retrospective propensity matched cohort study by Ranucci et al. [37]. In both studies, stimulation of erythropoiesis was associated with a reduction in RBC transfusion. 

A potential concern with ESA is the potential risk of thrombotic events observed in long-term treatment of patient with end-stage renal disease or cancer and in previous studies in spinal surgery without concomitant administration of antithrombotic prophylaxis [46] or in orthopedic surgery, where higher dose of erythropoietin were employed [47].

An increased risk of thrombosis with EPO in critically ill patients was observed in a large RCT including medical, trauma and surgical ICU patients [48]. Then, a post hoc analysis found that this increased risk was present only in patients who did not receive a baseline heparin treatment. 

In a recent meta-analysis, pooling 25 studies and about 5000 patients [49], the use of erythropoiesis stimulating agents did not increase the risk of myocardial infarction, stroke, deep venous thrombosis in the postoperative period. 

In agreement with previous RCTs [35,44,45] showing a safe profile of ESA in cardiac surgery, the two recent studies on anemia treatment in cardiac surgery by Ranucci and Spahn [36,37] did not report an excess of thrombotic events with erythropoietin.

It is also important to consider that the duration of ESA treatment and subsequent increase in Hb is limited to 3–4 weeks, further mitigating the risk of thrombosis. 

## 7. Optimizing Coagulation and Antithrombotic Treatment

### 7.1. Hemostatic Evaluation

The preoperative hemostatic evaluation of the patient is particularly important to detect any acquired or inherited hemostatic deficiencies in order to diminish the risk of perioperative bleeding complications by introducing an adapted treatment strategy if indicated. 

This evaluation includes the review of the medical history (history of transfusion, history of drug-induced coagulopathy, presence of congenital coagulopathy, history of thrombotic events, etc.), the interview of the patient to exclude hemorrhagic diathesis (for example by using the HEMSTOP score), the physical examination (ecchymosis, petechiae, pallor, etc.) and finally to conduct specific laboratory tests if indicated [5].

As recommended by the French Society of Anesthesia and Intensive Care in 2013, hemostatic laboratory testing should not be systematically requested in patients whose history and clinical examination do not suggest hemostatic disorders (recommendation Grade 1). The preoperative use of standard laboratory tests is also not recommended by the European Society of anesthesiology and Intensive Care in their last recommendations from 2017 [50]. First, the most common congenital hemostatic disorder, Von Willebrand Disease for example, is not detected by using the standard aPTT and PT tests, so normal standard hemostasis test results do not exclude the possibility of hemostatic disorders and resultant perioperative bleeding risk [51]. Second, the cost savings associated with rationalizing preoperative testing practice can be substantial [52].

The most important part of the assessment is based on the personal and family history of hemorrhagic diathesis and on physical examination [51] in order to guide the need of an in-depth assessment of hemostasis. Different questionnaires are available, like the ISTH bleeding assessment tool (BAT) [53], which can be used to guide the physician to objectively assess any bleeding symptoms. Less time consuming, the HEMSTOP questionnaire can be used to help the clinician to discriminate patients at an elevated risk for bleeding and require bleeding prophylaxis or precautions [54].

### 7.2. Discontinuation of Anticoagulants

The multiplication of the anticoagulants drugs in the last decade render the perioperative management more challenging. The timing of drug cessation will depend on the agents used, on their dosage and finally on specific patient characteristics, including renal function, hepatic function, age, body weight and finally on the presence of possible drug to drug interactions.

**Figure 2:**
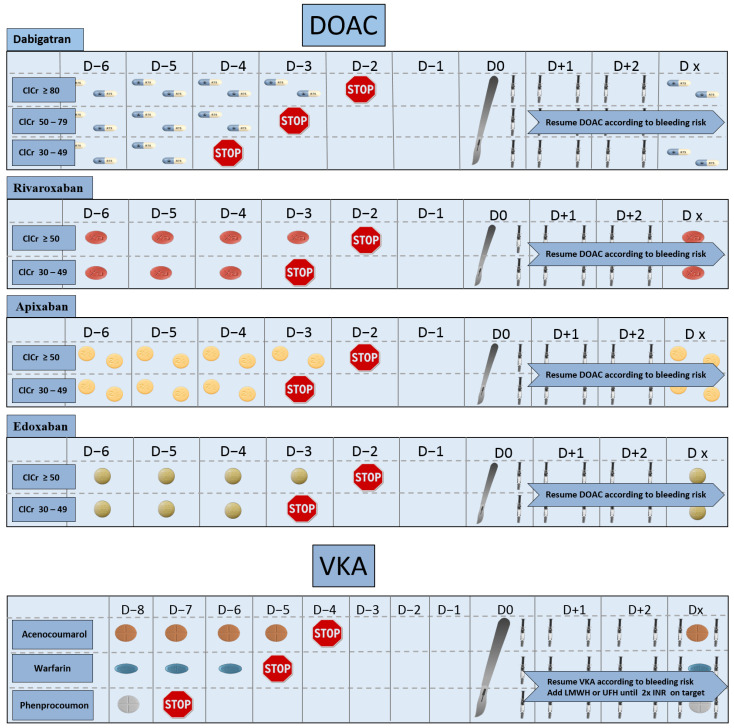
Our institutional scheme for management of anticoagulant medications in planned cardiac surgery. DOAC: direct oral anticoagulant, VKA: vitamin K antagonist, LMWH: low molecular weight heparin, UFH: unfractionated heparin; INR: international standardized ratio, ClCr: creatinine clearance (expressed in mL/min).

Cardiac surgery is considered as at high risk for bleeding and interruption of the anticoagulant agent must be timely to achieve a minimal residual anticoagulant activity at the time of surgery. Usually, a four-half-life delay between cessation and surgery is advocated. In our center, easy to interpret schemes are presented on wall posters in the surgical and anesthetic consultation rooms of the preoperative clinic. Figure 2 presents our institutional scheme for preoperative interruption and perioperative management of oral anticoagulants.

The usual delay to stop low molecular weight heparin (LMWH) and unfractionated heparin (UF) will depend on the dosage (therapeutic versus prophylactic), the molecule used and the route of administration (intravenous versus subcutaneous). In case of therapeutic dosing, the last dose of HBPM should be administered 24 h before surgery and intravenous UF should be interrupted 6 h before surgery [55].

For vitamin K antagonists (VKA), the time will depend on the specific agent used: the last dose of acenocoumarol should be taken 3 to 4 days before surgery, the last dose of warfarin 6 days before surgery and phenprocoumon should be interrupted up to 10 days prior to surgery [50,56]. 

For direct oral anticoagulants (DOAC), the evidence regarding the timing of interruption is low and predominantly based on the results of the PAUSE study [57] and pharmacokinetic assumptions [58]. The different guidelines [50,55,59,60,61,62,63] vary and question the clinician in daily practice [58].

For dabigatran, the last dose should be given 3 days before the surgery (skip 4 doses) in case of CrCl of >50 mL/min, and 5 days before the surgery (skip 8 doses) if CrCl of 30–49 mL/min [64]. More cautiously, the EHRA recommended in 2018, before the PAUSE study results, to interrupt dabigatran 72 h or more in case of high risk bleeding procedure and a CrCl of 50–79 mL/min [63].

For anti-Xa agents, the last dose should be given 3 days before surgery if the CrCl is >30 mL/min. [64] The latest recommendations do not recommend any adaptation in case of renal impairment, contrary to some previous recommendations where earlier interruption was advocated for a Creatinine Clearance of less than 30 mL/min. This attitude can be questionable, particularly for edoxaban which has a 50% renal elimination [65].

In 2015, Godier et al., investigated the timing of interruption and showed that “a 48 h discontinuation of dabigatran or rivaroxaban did not guarantee a minimal DOAC concentration for all patients”, suggesting that a longer interruption should be safer in planed invasive procedure with a high hemorrhagic risk. Ref. [66] The author referred again to the known high interindividual pharmacokinetic variability [67] of rivaroxaban and dabigatran, illustrated in this study through the wide range and unpredictability of measured residual DOAC concentrations after the cessation of the drug, particularly within the first 24 h. 

The PAUSE study subanalysis from Shaw [68] in 2020 was performed to identify clinical parameters associated with residual DOAC levels of more than 30 ng/mL. Older age, female sex, low weight, renal dysfunction and shorter interruption were associated with risk of elevated preprocedural DOAC levels and the authors concluded that further study is required to determine whether adjustments to perioperative interruption based on these clinical parameters could result in a lower risk of residual DOAC levels. In case of possible drug accumulation (renal dysfunction, low body weight, older age and/or drug to drug interaction), the time interval should be increased and the anti-Xa or anti-IIa activity should be measured in particular cases [63].

In our opinion, renal function should be considered, particularly for edoxaban, which has a 50% renal elimination and was not investigated in the PAUSE trial. Therefore, the recommended timing might not be suited for this xaban specifically. However, there is no evidence, nor recommendations, supporting our opinion. 

If DOAC laboratory testing is used, a residual DOAC plasma concentration < 30 ng/mL would confirm the absence of significant anticoagulant effect [69] and it is accepted that high risk surgery can be performed without increased bleeding risk [66]. Unfortunately, there is a lack of evidence for any threshold to predict increased bleeding.

### 7.3. Discontinuation of Antiaggregants

Dual antiplatelet therapy is the gold standard for treatment of acute coronary syndromes and for the prevention of stent thrombosis after percutaneous coronary interventions. An increasing number of patients scheduled for cardiac surgery are treated with aspirin combined to a P2Y12 receptor inhibitor considerably increasing their risk of postoperative bleeding. Clopidogrel is associated with a tenfold increase in risk of reoperation for bleeding after CABG, and prasugrel increases bleeding by 400% compared to clopidogrel [70,71].

**Figure 3:**
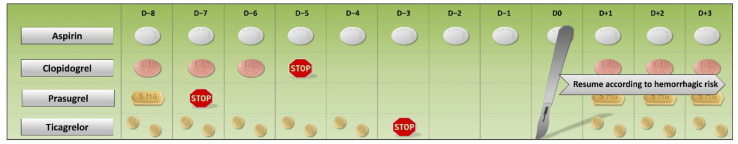
Our institutional schema for management of antiplatelet medications in planned cardiac surgery.

Recommended preoperative interruption delays for the various drugs are 7 days for prasugrel, 5 days for clopidogrel and 3 days for ticagrelor (recommendation IIb, level B) [72]. Figure 3 presents our institutional scheme for the perioperative management of antiaggregant drugs. 

However, important interindividual variation in the recovery of platelet function after cessation of the inhibitor is well documented. Up to 30% of patients show some degree of resistance to clopidogrel. [73] In case of high on treatment platelet reactivity, the premature interruption of the antiplatelet drug can put the patients of risk of coronary thrombosis. The degree of platelet inhibition can be easily tested by several easy to perform laboratory or point of care tests (e.g., TEG^®^ platelet mapping (Niles, IL, USA), Multiplate^®^ (Mannheim, Germany), PFA^®^ (Munich, Germany). Several studies show that personalized interruption of the antiplatelet drug prior to surgery based on monitoring of the degree of platelet inhibition can reduce preoperative waiting times without increasing bleeding risk. Refs. [74,75,76] Although convincing, operation schedules often do not allow for the degree of flexibility necessary to program elective interventions on a last day notice. Additionally, due to the paucity of data available at this time, preoperative platelet function testing to determine the time of surgery only is a grade IIb level B recommendation [72].

## 8. Challenges in the Intra-Operative Phase

In the cardiac operating theatre three actors work together: the surgeon, the anesthetist and the perfusionist. The actions of one will impact the others. Thus, recommendations for intraoperative measures should not be categorized by discipline but presented as single bundle.

This multidisciplinary team is responsible for the intraoperative phase of patient blood management and sharing a common culture and the same objectives is fundamental in improving patient’s outcome. 

The following paragraph resumes the intraoperative measures of PBM recommended in the 2019 joint guidelines [77] on cardiopulmonary bypass in adult cardiac surgery form the European Association for Cardiothoracic Surgery (EACTS), the European Association of Cardiothoracic Anesthesiologists (EACTA) and the Board of Cardiovascular Perfusionists (EBPC) and the 2017 EACTA/EACTS guidelines [7] on patient blood management for adult cardiac surgery. 

### 8.1. Anesthetic Management

Basic favorable conditions for coagulation such as temperature >36 °C, pH (nearly 7.4) and normal calcium levels need to be maintained outside of the CPB phase.

Routine use of antifibrinolytics, and limitation of hemodilution by fluid restriction are standard measures that can easily be applied to most, if not all, patients. Avoidance of vasodilation by excessive sedation, and the use of vasoconstrictors to counteract vasodilation secondary to general anesthesia reduces intravascular volume depletion. Limiting blood sampling to only answer relevant questions reduces blood loss. 

### 8.2. Management of Shed Blood and Cell Savage

Shed blood, coming from cardiotomy, pleural and pericardial suction contains inflammatory mediators, activated platelet and coagulation factors and might be responsible for thrombin generation, pro-fibrinolytic and inflammatory pathways activation. Activation of shed blood is more pronounced if the blood is left to stagnate in the chest cavity. Depending on the volume, prompt suction with a separate line for shed blood is recommended whenever possible. Shed blood could be processed with a cell saving device before returning to the patient. 

The processing of too large volumes (>1000 mL) through a cell saver may have negative effects on coagulation, since plasma and coagulation factors are lost [7].

### 8.3. Minimal Invasive Extracorporeal Circuits (MiECC)

A minimally Invasive Extra Corporeal Circuit combines several recent advances in CBP technology. 

MiECC is characterized by a completely closed circuit, void of blood-air interfaces, with biologically inert contact surfaces (coated tubing), small priming volume (short tubing), a centrifugal pump, a separate shed blood management system with cell savage and the absence of cardiotomy suction in the heart-lung machine [78]. 

The use of MiECC systems hypothetically leads to a reduction in hemodilution and postoperative bleeding, resulting in less RBC transfusion and re-exploration, but definitive proof is lacking. This potential benefit could be more pronounced in small-size adults or in patient refusing transfusions [79]. 

Others potential benefits are attenuation of inflammatory response, lower incidence of stroke, atrial fibrillation and renal failure, improved myocardial protection, improved microvascular organ perfusion with subclinical evidence for protective effects on lung, liver and intestine. 

Actual evidence on MiECC comes from meta-analysis of small RCTs, underpowered to detect an impact on mortality. The largest meta-analysis including 2700 patients from 24 RCTs suggests a possible reduction on mortality in CABG surgery [80]. 

### 8.4. Autologous Priming 

Crystalloid priming of a CBP circuit results in hemodilution and therefore increases the risk of blood transfusion. With the autologous priming technique, at initiation of CPB, the crystalloid solution that fills the circuit is evacuated and replaced by the patient’s blood. 

Autologous priming is a safe technique that should be considered as a part of a blood conservation strategy (Class IIa, LOE A 2017 EACTA Guidelines on PBM). 

### 8.5. Heparin/Protamine Management

Unfractionated heparin is the anticoagulant of choice for cardiac surgery using CPB, and protamine is the antidote administered after CBP weaning. With ACT based algorithms the optimal protamine to heparin ratio is 0.8–1 of the initial dose of heparin. Yet, the protamine–heparin dose–response curve shows great interindividual variation and patient tailored protamine dosing reduces residual heparin effects as well as protamine overdosing.

Inappropriately high doses of protamine in absence of heparin are associated with enhanced postoperative bleeding and need for transfusion [77]. 

In some studies [81,82,83,84,85], individual heparin and protamine titration using an automated heparin titration device (Hepcon^®^ Hemostasis Management System, Medtronic, Minneapolis, MN, USA) was associated with decreased blood loss and transfusion requirements compared to a standard ACT management. However, bleeding and transfusion were secondary end points in these studies and larger multicenter studies are required [77].

Generally when an HMS^®^ is used, higher heparin and lower protamine doses are administered, in both valvular [86] and CABG surgery [87], with a better preservation of platelet function and coagulation. 

In patients with contraindications to heparin and/or protamine usage, such in case of protamine allergy or heparin induced thrombocytopenia, bivalirudin is an alternative for anticoagulation in patients without severe renal function impairment, since about 20% of the drug is cleared unchanged by the kidney. According to EACTS/EACTA/ECCP guidelines, a baseline ACT value should be measured before bivalirudin administration and a target of an ACT of 2.5 times baseline is recommended during CPB [77].

### 8.6. Targeted Treatment of Coagulopathies

After antagonization of heparin, ongoing bleeding is frequent and should be treated aggressively by meticulous surgical hemostasis and targeted treatment of coagulopathies. Residual effects of anticoagulants or antiaggregants taken preoperatively can be hard to treat and prothrombin complex concentrates, or platelet transfusion are often indicated. Specific antidotes for the direct oral anticoagulants are becoming available but solid evidence of their clinical utility is lacking and their prices can be prohibitive. For the irreversible P2Y12 inhibitors clopidogrel and prasugrel, platelet transfusion can restore platelet function. Ticagrelor, on the other hand has a reversible bond with the receptor and will be evenly distributed over all the receptors available, including those of freshly transfused platelets. A recent find is that the hemadsorption device Cytosorb^®^ can readily remove apixaban, rivaroxaban and ticagrelor for circulation. The device can be integrated into the CPB circuit, resulting in at least partial elimination of the drugs by the time the patient is separated from bypass.

Targeted treatment of hemostatic disorders using point of care viscoelastic test is increasingly popular and has a growing body of evidence showing reduced bleeding and transfusion requirement. Several devices, based on different technologies are commercially available of which TEG^®^ and ROTEM^®^ are the most used and best studied [88]. Despite the obvious theoretical advantages of rapidly identifying and treating specific hemostatic deficiencies, besides reducing exposure to blood products, the large majority of studies did not find a benefit on mortality or morbidity [89,90,91,92]. 

### 8.7. The Use of Factor Concentrates in Cardiac Surgery

FFP contains all the coagulation factors, fibrinogen and coagulation inhibitors in physiologic concentrations. Increasingly, purified factor concentrates, rather than FFP, are used to substitute factor deficiencies after cardiac surgery. Ref. [93] The advantages of using factor concentrates are the immediate availability, the absence of ABO incompatibility and their sterile, virus deactivated fabrication. The commercially available factor concentrates used can be divided in four groups: fibrinogen concentrates, four factor prothrombin complex concentrates and recombinant activated Factor VII.

Of these, fibrinogen concentrate is the best studied, yet conclusive evidence remains limited, and recommendations are class IIb, level B in the 2017 EACTA guidelines. A 2018 metanalysis, however, has shown fibrinogen to significantly reduce transfusion requirements without or thrombosis related morbidity or mortality. Ref. [94] A 2019 EACTA consensus statement recommends fibrinogen concentrate to treat microvascular bleeding after cardiac surgery, based on its efficacy and good safety profile. Ref. [95] In our practice, fibrinogen concentrate has become the first-choice treatment for bleeding after cardiac surgery.

Four factor prothromplex complex concentrates, or PCCs contain the vitamin K dependent coagulation factors as well as inhibitors such as heparin and proteins S and C. They have a high thrombin generation potential and may therefore lead to a higher incidence in thrombotic events. The sparse data available does not confirm this. A retrospective study compared two time periods before and after the change form an FFP/platelet-based protocol for bleeding to a PCC/platelet-based protocol. Ref. [96] A total of 118 matched patients were compared for transfusion requirements, postoperative blood loss, the need for reintervention, duration of postoperative ventilation, acute kidney injury, stroke, new onset atrial fibrillation, postoperative myocardial infarction, thromboembolic events, infectious complications, length of stay and 30-day mortality. 

Blood loss, RBC units transfused and the number and volume of FFP transfused were significantly lower in the PCC patients. There was no difference in the need for platelet transfusion, and importantly, there was no increase in thromboembolic events, stroke or perioperative infarction. Although encouraging, the lack of data and the theoretical risk of thrombosis warrants caution in the use of PCC until more studies come available. 

Recombinant activated factor VII increases the incidence of major thromboembolic events without reducing transfusion rates and is therefore not recommended in cardiac surgery except for patients suffering uncontrolled refractory life-threatening hemorrhage [7,97].

### 8.8. Temperature

The negative impact of hypothermia on coagulation has been known for four decades. Patients undergoing hypothermic CPB had increased blood loss and surgical re-exploration [98].

Hypothermia negatively effects both primary and secondary hemostasis. Hypothermia induces contradictory changes in platelets such as increased marginalization and longer platelet half-life. Platelets are primed for activation and hypothermia-induced changes in the structure of the vWF-receptor make them more vulnerable to macrophage phagocytosis [99]. During hypothermic cardiac surgery, platelet function is suppressed and only partially recovers after rewarming. Ref. [100] The inhibition of enzymatic reactions of the coagulation cascade was demonstrated by prolongation of prothrombin time and partial thromboplastin time tests in hypothermic situations, when factor levels were all known to be normal [101].

In vitro studies, using whole blood and measurements by thromboelastography, show that initiation, propagation of coagulation and stability of blood clot are impaired by hypothermia and authors recommend to assess clot formation at the patient’s body temperature, in order to avoid over-estimation of coagulation performance [102].

While patient cooling during CPB was standard practice up to a decade ago, selective cooling of the myocardium through cold cardioplegia solutions while maintaining normal body temperature has become the new standard. Refs. [103,104] In some highly complex cardiac procedures, such as aortic arch repair, hypothermia remains the gold standard for brain protection. Nevertheless, normothermic techniques for even total arch replacement have been developed and seem safe [105].

The use of normothermic techniques is recommended to reduce transfusion requirements in cardiac surgery (recommendation class IIb level of evidence B) [7].

### 8.9. Off-Pump Surgical Revascularization

Off-pump coronary artery bypass grafting (OPCABG) is an alternative surgical technique for myocardial revascularization that avoids CPB, great vessels cannulation, aortic cross-clamping and cardioplegia. Anastomosis are performed on a beating heart, with target sites being stabilized by specialized equipment. Lower doses of systemic heparin are generally required, about one third that used for CPB, i.e., 100–150 IU/kg [106].

Three recent meta-analyses compared OPCABG to on-pump CABG, including more than 100 RCTs [107,108,109]. Bleeding related outcomes were secondary endpoints in all these RCTs, so that no definitive recommendations can be derived. 

In 2015, Puskas et al. [108] performed the largest meta-analysis including 19,101 patients with different surgical risk. The rate of RBC transfusion was lower in OPCABG group (OR 0.49, CI 95% 0.33–0.72), and there was no difference in rate of surgical re-exploration for bleeding (OR 0.99, CI 95% 0.74–1.34). These results were confirmed in 2016 by Deppe et al. [107], showing a decrease in the number of units of transfused RBC in patients operated without the use of CPB, with a similar rate of re-sternotomy for bleeding. 

In a more recent meta-analysis, Sheafi et al. [109] compared OPCABG vs. on-pump CABG regarding short- and long-term outcomes. The two larger high quality RCTs included in these meta-analysis, CORONARY and GOBCAPE trials [110,111], suggest that OPCABG does not offer a long-term benefit on mortality or cardiac complications, but it may be associated with lower transfusion rates (50.7% vs. 63.3% and 56.3% vs. 62.7%, respectively; both *p* < 0.01). 

Therefore, important considerations in patient selection for OPCABG should include not only bleeding risk and the presence of atheromatous disease of ascending aorta but also other aspects that may negatively impact long-term surgical and clinical results, such as the surgeons experience, small size of coronary arteries, intramyocardial vessels, low ejection fraction carrying the risk of intraoperative hemodynamic instability leading to incomplete revascularization. 

### 8.10. Acute Normovolemic Hemodilution

Acute normovolemic hemodilution is the practice in which a certain volume of whole red blood is drawn from the patient, after the start of anesthesia, and stored in one or two collection bags containing a citrate–phosphate–dextrose anticoagulant. The loss in circulating volume is compensated with colloids or crystalloids. This technique is reserved for predictably hemorrhagic interventions such as cardiac surgery. The bags are set aside and re-transfused to the patient when the hemorrhagic episode is over. The rationale for this technique is that red blood cell mass, plasma and platelets are harvested before bleeding and that the patient’s own whole blood can be used to restore the circulating volume, the red blood cell mass and the hemostatic capacity. The hypothetical advantage of this technique is that transfusion reactions to allogenic blood products are likely to be avoided and that whole blood may have superior hemostatic capacity than fractioned blood products. Specifically for cardiac surgery, ANH protects the withdrawn blood from the detrimental effects of CPB [112].

Practically, the collection bag needs to be connected to a large bore catheter inserted in a proximal vein (e.g., antecubital vein) or to a large bore central venous catheter. The collection bag is then lowered to allow for drainage by gravity. This relatively simple technique comes, in our experience, with some hidden flaws that the anesthesia team must be aware of. First, placing the bag on the floor seems unhygienic and puts it at high risk of being stepped on, since the space occupied by the anesthesia team can be quite crowded. The collection bag should therefore be rested on a step which can be placed underneath the operating table. Second, the blood and anticoagulant need to be adequately mixed by frequent manual kneading or by an automated mixing device. Third, the anticoagulant capacity or the citrate–phosphate–dextrose mixture can be exceeded by overfilling of the bag. It is very hard to visually evaluate the volume withdrawn and a more accurate estimate can be made by placing the bag on a balance. Both inadequate mixture and overfilling of the bag can lead to cloth formation in the bag, and loss of the whole-blood product. Ref. [113] Finally, the loss of red blood cell mass due to ANH and the subsequent hemodilution during CPB can lead to unacceptable low hemoglobin values during CPB. This technique can therefore only be used safely in patients with normal to high preoperative hemoglobin levels.

Barile et al. [114] compiled the results of 29 randomized controlled trials investigating the effects of ANH (*n* = 1252) versus control (*n* = 1187) in cardiac surgery on transfusion requirements and blood loss. Patients in the ANH arms were overall less likely to receive allogenic blood products (relative risk of 0.74), had lower exposure to allogenic products when transfused (mean difference = −0.79 units of RBC per patients), and had statistically significant less blood loss (388 mL vs. 450 mL, *p* < 0.0001). The limited clinical benefit led the conclusion that ANH may be, at best, considered as an adjunct technique to PBM with a class IIB recommendation.

## 9. Postoperative Management: Applying Appropriate RBC Transfusion Triggers

The decision to transfuse blood products should be guided by careful evaluation of the patient’s clinical rather than by a fixed hemoglobin level.

Anemia becomes dangerous when oxygen delivery does not match oxygen metabolic demand. Determinants of oxygen delivery are cardiac output and arterial oxygen content. Hemoglobin is a crucial factor in determining the latter. The physiological response to anemia is an increase in cardiac output, which can be compromised in post cardiac surgical patients. 

Signs of adequacy of oxygen delivery/consumption must be carefully evaluated. SVO2 and blood lactate monitoring, myocardial ischemia, end-organ ischemia, low cardiac output syndrome, inotropes or vasopressors requirements and diuresis.

Several trials have tried to clarify the unanswered question whether a more liberal transfusion strategy of RBCs after cardiac surgery improves the oxygen balance and results in better outcomes. Two RCTs showed that restrictive transfusion strategies are non-inferior to liberal strategies. Hajjar et al. [115] randomized 512 patients into a liberal group with a transfusion trigger of a hematocrit less than 30% and a restrictive group with a trigger at a hematocrit less than 24%. Mazer et al. [116] randomized 5243 patients into a group with a transfusion threshold set at 9.5 g/dL of hemoglobin and a group with the threshold set at 7.5 g/dL. Both studies found no difference for the primary outcome measure, a composite of all-cause 30-day and, respectively, 28-day mortality or severe morbidity and concluded that a restrictive strategy is non-inferior to a liberal strategy. Finally, in the debated study by Murphy et al. [117], where 2007 patients were divided in a liberal (Hb less than 9 g/dL) and a restrictive group (Hb less than 7.5 g/dL), the primary outcome, a combination of infectious or ischemic events was reached in similar numbers of patients, but 90-day mortality was significantly higher in the restrictive group. The absolute difference was an increased mortality rate of 1.6% and just reached statistical significance (*p* < 0.045). In the absence of a difference in any of the other morbidity outcomes (wound infection, sepsis, stroke, myocardial infarction, gut infarction and acute kidney injury) the increased mortality is hard to interpret. The authors concluded that a restrictive transfusion strategy is non-superior to a liberal one.

In a recent metanalysis including 13 randomized trials comparing restrictive transfusion strategies (*n* = 4545) with liberal strategies (*n* = 4547), the pooled data showed no difference in 30-day mortality, myocardial infarction, renal failure, infection or arrythmia [118]. Mortality was 2.9% in the restrictive group and 3% in the liberal group. These mortality rates are similar to those reported in anemic patients undergoing cardiac surgery by other authors and are invariably higher than for non-anemic patients [29,119].

The conclusion that almost inevitably presents itself when interpreting this data is that preoperative anemia puts the patient in a particularly bad position for the surgery he or she is about to undergo, and transfusion of RBC to maintain a hypothetical oxygen delivery capacity does next to nothing to improve their odds. 

## 10. Conclusions

After decades of research, a picture starts to emerge. When an anemic patient goes into cardiac surgery or if anemia installs during surgery, the patient is shifted to a lower survival curve. Counterintuitively, transfusion of RBCs will in many patients not restore the odds of survival. All efforts should therefore be made to detect and treat preoperative anemia and to prevent blood loss during and after surgery. The concept of patient blood management is based on the combination of interventions and practices which all serve that purpose. The creation and sustainability of a PBM program requires a high level of organization and collaboration form a motivated, convinced multidisciplinary team. A reduction in the use of blood products will be the earliest and clearest result, and maybe the only one directly measurable in the short term. Nevertheless, despite the challenges a PBM program may pose, we are convinced that the shoulder shrugging attitude in the face of anemia is, or must be, a thing of the past and that with a combined effort patient care can be brought to a much higher level.

## Figures and Tables

**Table 1:** Summary of our recommendation for preoperative, intraoperative and postoperative phases of Patient Blood management in cardiac surgery.

Preoperative patient blood management for cardiac surgery	
Identify and correct anemia to optimize red blood cells mass	Perform lab test if expected blood loss > 500 mL or Transfusion risk > 10%; Consider intravenous iron supplementation if an absolute iron deficiency is present irrespective the presence of anemia; Consider intravenous iron supplementation in case of anemia due to inflammatory disease and chronic heart failure; Consider EPO and iron supplementation in case of anemia due to chronic renal failure.
Optimize coagulation	Perform a hemostatic evaluation with focus on personal and family history and physical examination; Discontinue anticoagulant drugs; ○ Acenocoumarol 3–4 days before surgery; ○ Warfarin 5 days; ○ Phenprocoumon 7 days; ○ Discontinue DOAC (Dabigatran, Rivaroxaban, Apixaban, Edoxaban) at least 48 h before surgery; ○ For DOAC apply longer discontinuation times according to creatinine clearance to achieve 4 half-lives (i.e., up to 96 h); Discontinue antiplatelet drugs (except Aspirin); ○ Clopidogrel 5 days before surgery; ○ Prasugrel 7 days; ○ Ticagrelor 3 days.
Intraoperative patient blood management for cardiac surgery	
Optimize coagulation	Maintain body temperature > 36 C, normal pH and calcium level; Use antifibrinolytics; Avoid hemodilution, excessive sedation, unnecessary blood sampling; Use Cell savage; Consider point of care viscoelastic test.
CPB management	Consider minimally invasive extracorporeal circuits (MiECC); Consider autologous priming; Consider individual heparin and protamine titration using automated heparin titration devices; Consider ultrafiltration.
Surgical management	Consider Minimal invasive techniques; Consider OPCABG over on-pump CABG in selected cases; Avoid stagnation of shed blood in the chest cavity.
Postoperative patient blood management in cardiac surgery	
Harness anemia tolerance Apply appropriate transfusion triggers	Optimize oxygen delivery; Reduce oxygen consumption: optimal pain control, avoid tachycardia and hypertension; Continue to treat anemia; Transfuse if Hb < 7 g/dl or Hct < 21%; Avoid unnecessary transfusion (i.e., “top up” RBC transfusions).

## Data Availability

Not applicable.
